# Evaluation of the Effects of Atorvastatin and Ischemic Postconditioning Preventing on the Ischemia and Reperfusion Injury: Experimental Study in Rats

**DOI:** 10.21470/1678-9741-2017-0108

**Published:** 2018

**Authors:** Henrique Budib Dorsa Pontes, José Carlos Dorsa Vieira Pontes, Euler de Azevedo Neto, Giovanna Serra da Cruz Vendas, João Victor Cunha Miranda, Letícia do Espírito Santos Dias, João Victor Durães Gomes Oliva, Murilo Henrique Martins de Almeida, Ian de Oliveira Chaves, Tricia Luna Sampaio, Carlos Henrique Marques dos Santos, Doroty Mesquita Dourado

**Affiliations:** 1 Universidade Anhanguera (Uniderp), Campo Grande, MS, Brazil.; 2 Universidade Federal do Mato Grosso do Sul (UFMS), Campo Grande, MS, Brazil.

**Keywords:** Reperfusion Injury/Prevention & Control, Hydroxymethylglutaryl-CoA, Oxidative Stress, Ischemic Postconditioning, Rats, Wistar

## Abstract

**Introduction:**

Reperfusion injury leads to systemic morphological and functional pathological alterations. Some techniques are already estabilished to attenuate the damage induced by reperfusion. Ischemic preconditioning is one of the standard procedures. In the last 20 years, several experimental trials demonstrated that the ischemic postconditioning presents similar effectiveness. Recently experimental trials demonstrated that statins could be used as pharmacological preconditioning.

**Methods:**

41 Wistar rats (*Rattus norvegicus albinus*) were distributed in 5 groups: Ischemia and Reperfusion (A), Ischemic Postconditioning (B), Statin (C), Ischemic Postconditioning + Statins (D) and SHAM (E). After euthanasia, lungs, liver, kidneys and ileum were resected and submitted to histopathological analysis.

**Results:**

The average of lung parenchymal injury was A=3.6, B=1.6, C=1.2, D=1.2, E=1 (*P*=0.0029). The average of liver parenchymal injury was A=3, B=1.5, C=1.2, D=1.2, E = 0 (*P*<0.0001). The average of renal parenchymal injury was A=4, B=2.44, C=1.22, D=1.11, E=1 (*P*<0.0001). The average of intestinal parenchymal injury was A=2, B=0.66, C=0, D=0, E=0 (*P*=0.0006). The results were submitted to statistics applying Kruskal-Wallis test, estabilishing level of significance *P*<0.05.

**Conclusion:**

Groups submitted to ischemic postconditioning, to pre-treatment with statins and both methods associated demonstrated less remote reperfusion injuries, compared to the group submitted to ischemia and reperfusion without protection.

**Table:** 

Abbreviations, acronyms & symbols				
ADP	= Adenosine diphosphate		HMG-CoA reductase	= 3-hydroxy-3-methylglutaryl-coenzyme A reductase
ALT	= Alanine transaminase		IL-1	= Interleukin-1
AST	= Aspartate transaminase		IL-6	= Interleukin-6
ATP	= Adenosine triphosphate		mRNA	= Messenger ribonucleic acid
cAMP	= Cyclic adenosine monophosphate		NADPH	= Nicotinamide adenine dinucleotide phosphate
eNOS	= Endothelial nitric oxide synthase		NO	= Nitric oxide
EROs	= Reactive species of oxygen		OONO	= Peroxynitrite
ET-1	= Endothelin-1		ROS	= Reactive oxygen species
FPP	= Farnesyl pyrophosphate		TNF-alpha	= Tumor necrosis factor alpha
GGPP	= Geranylgeranyl pyrophosphate			

## INTRODUCTION

### Ischemia and Reperfusion Lesion, Inflammation and Oxidative Stress

In an experimental study in rats in 1986, Parks and Granger^[1]^ demonstrated that the mucosal injury membrane was more severe in rats submitted to 3 hours of ischemia followed by 1 hour of reperfusion than that produced by exclusive ischemia for 4 hours. Since then, it has been proposed that reperfusion has more damaging effects on cellular structures than ischemia itself. This event was called ischemia and reperfusion injury.

The ischemia and reperfusion injury has its mechanisms closely related to the inflammatory response and the production of reactive oxygen species (ROS). During the ischemic period, the cell enters in an anaerobic metabolism, and the oxidative phosphorylation of the mitochondria does not occur, so glycolysis assumes the leading role in the production of adenosine triphosphate (ATP), but it is not enough. During ischemia, ATP degradation in adenosine diphosphate (ADP), cyclic adenosine monophosphate (cAMP), adenosine, inosine and finally the accumulation of hypoxanthine occurs, which during reperfusion enhances ROS production by the enzyme xanthine oxidase. The lower availability of ATP leads to changes in the ATP-dependent ion exchange pumps, leading to intracellular accumulation of sodium and ultimately to cell swelling and the entry of calcium ions. The calcium entry into the intracellular medium leads to cytoskeleton contraction, activation of phospholipase A2, and release of arachidonic acid and its metabolites. From the degradation of the arachidonic acid products, pro-inflammatory mediators, such as prostaglandins, leukotrienes and troboxanes, which activate neutrophil adhesion and infiltration, and platelet aggregation, are activated. Once in the tissues, neutrophils produce large amounts of ROS leading to oxidative stress^[2]^.

**Fig. 1:**
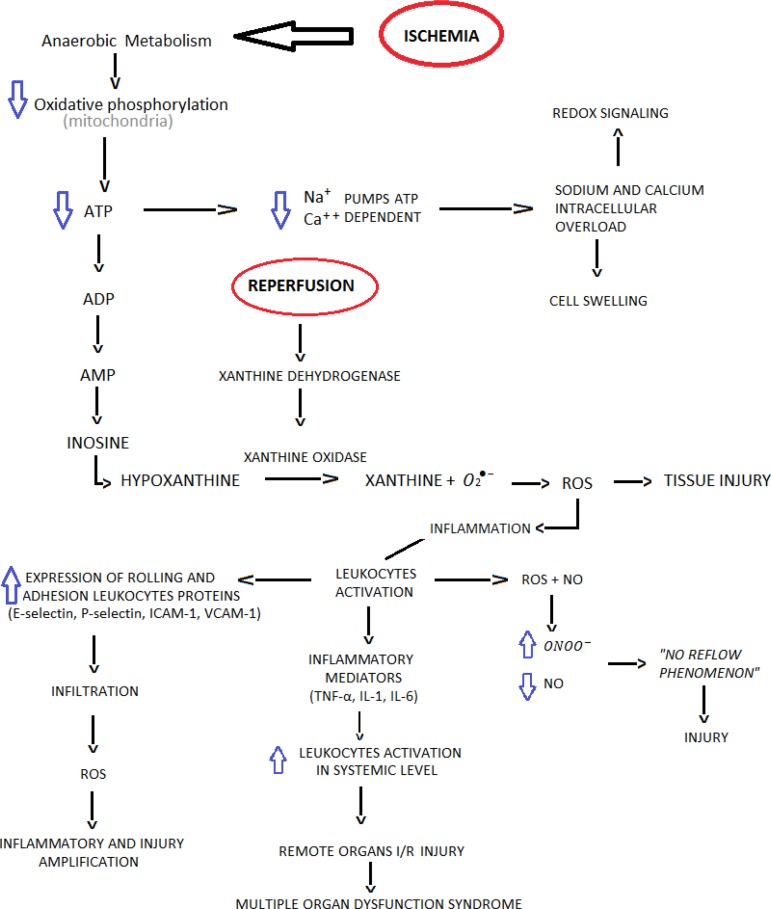
Mechanism of ischemia-reperfusion and oxidative stress. I/R=ischemia/reperfusion, ADP=adenosine diphosphate; AMP=adenosine monophosphate; ATP=adenosine triphosphate; IL-1=Interleukin-1; IL-6=Interleukin-6; NO=nitric oxide; OONO-=Peroxynitrite; ROS=reactive oxygen species; TNF-α=tumor necrosis factor alpha

The ROS overload leads to a decrease in nitric oxide (NO) bioavailability, reacting with NO leading to the production of peroxynitrite (OONO-), a potent oxygen-reactive species, highly damaging to cells. Decreased availability of NO leads to a redistribution in circulation during the reperfusion period, the socalled non-reflux phenomenon, leading to tissue damage. During the ischemia and reperfusion injury, massive quantities of proinflammatory mediators such as tumor necrosis factor alpha (TNFalpha), interleukin-1 (IL-1) and interleukin-6 (IL-6) are released into the bloodstream, leading to neutrophilic activation and amplification of the pro-inflammatory response at the systemic level, thus reaching organs that were not submitted to direct ischemia and could lead to multiple organ dysfunction syndrome^[2]^ (Figure 1).

### Ischemic Preconditioning and Ischemic Postconditioning

From the discovery of the ischemia and reperfusion injury, several methods were proposed to try to attenuate the lesion during reperfusion. Murry et al.^[3]^, in 1986, proposed the ischemic preconditioning method, which consists of subjecting a tissue to short cycles of ischemia and reperfusion before submitting it to a prolonged time of ischemia, demonstrating the benefit of this technique in rats isolated arteries. However, it is not always possible to apply this technique, since in some conditions - such as pulmonary thromboembolism, mesenteric ischemia and acute myocardial infarction - ischemia is already present. For this purpose, the technique of ischemic postconditioning was proposed, which consists in subjecting the tissue to short cycles of reperfusion and ischemia after the period of ischemia and before subjecting it to reperfusion for a prolonged period. Santos et al.^[4]^ demonstrated that the efficacy of this method is similar to the efficacy of ischemic preconditioning, and there is no statistically significant difference between the two. This technique is also able to protect tissues at a distance and can be applied in arteries distal to the primary ischemic process, which is effective as demonstrated by Dorsa et al.^[5]^, and was thus called remote postconditioning.

Since the discovery of the methods of minimization of ischemia and reperfusion injury and its involvement with oxidative stress, alternatives to drug use, the so-called pharmacological conditioning, are sought, with the advantage of not manipulating vessels, thus being non-invasive. Among the drugs capable of producing this effect, antioxidants and immunomodulators stand out.

### Statins as Potential Anti-Inflammatory and Modulation of Oxidative Stress

Statins are drugs widely used around the world. It is estimated that about 30 million people use some statin. This medication is used as an antidislipidemic, and its mechanism of action is to inhibit the enzyme 3-hydroxy-3-methylglutaryl-coenzyme A reductase (HMG-CoA reductase), a key enzyme in liver cholesterol biosynthesis responsible for catalyzing the reduction of HMG-CoA in mevalonate^[6]^.

**Fig. 2:**
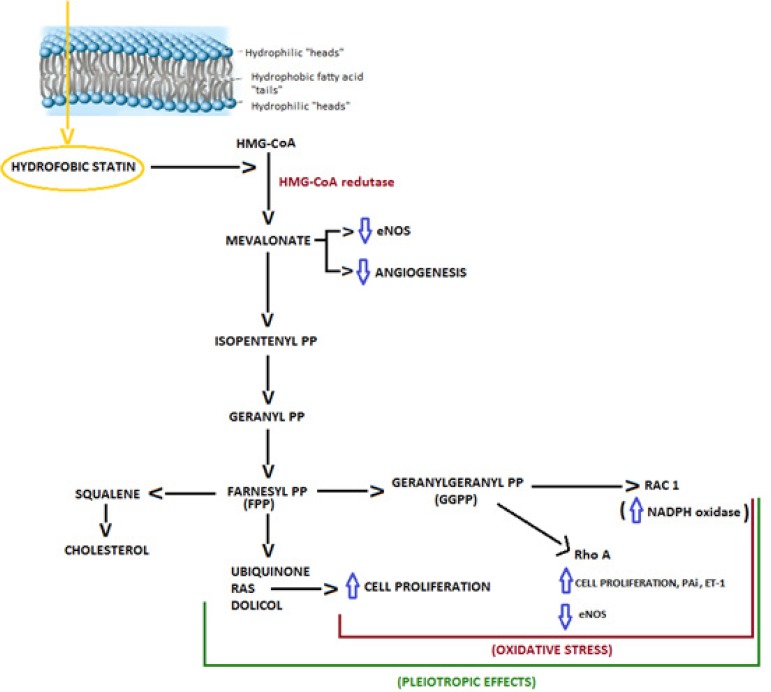
Action mechanism of the statins.

However, statins seem to exert independent effects of cholesterol, called pleiotropic effects. By inhibiting the conversion of HMG-CoA to L-mevalonate, statins prevent the synthesis of isoprenoids, which are precursors of cholesterol biosynthesis, which serve as important lipid ligands for post-translational modification of intracellular proteins, in particular farnesyl pyrophosphate (FPP) and geranylgeranyl pyrophosphate (GGPP), which are responsible for the isoprenylation of wide-range OS proteins as small proteins associate with guanosine triphosphate such Rho, Rac and Ras GTPases. This protein isoprenylation allows adequate subcellular localization and intracellular trafficking of proteins, which control various cellular functions, and the inhibition of these pathways may determine important components of the pleiotropic effects of statins. The Rho protein pathway is related to oxidative stress, atherosclerosis and elevated blood pressure. The Rac protein pathway signaling is involved in two crucial mechanisms, such as cytoskeletal remodeling and ROS synthesis. Regarding the production of reactive species of oxygen (EROs), Rac1 binds to p67phox leading to the activation of the nicotinamide adenine dinucleotide phosphate (NADPH) oxidase system, and subsequent generation of EROs. In addition, inflammatory cytokines are mediated by Rac^[7]^ (Figure 2).

Statins also appear to improve endothelial function by both cholesterol-dependent and cholesterol-independent mechanisms. Some studies have shown an improvement in endothelial function independent of the reduction of serum cholesterol levels^[8]^.

Statins increase endothelial nitric oxide synthase (eNOS) expression, more by prolonging the half-life of messenger ribonucleic acid (mRNA) than by induction of eNOS gene transcription, which under conditions of oxidative stress can become uncoupled and produce O2 instead of NO contribuiting for endotelial disfunction. Another important effect is the reduction of the abundance of caveolin-1, an eNOS binding protein, which directly inhibits NO production, thus increasing the availability of this vasodilator. Statins also demonstrated the ability to increase the expression of T-PA and inhibition of endothelin-1 (ET-1) expression^[9]^. These mechanisms suggest that inhibition of these intermediary pathways may contribute to the pleiotropic effects of statins, minimizing ROS production and improving vascular and endothelial function^[8]^.

This work was carried out in order to achieve responses regarding the efficacy of statin in minimizing or even preventing ischemia and remote reperfusion injury compared to the established ischemic postconditioning method and the efficacy of these associated methods.

## METHODS

### Animals

An experimental study was carried out in rats at the laboratory of the Agrarian campus at Universidade Anhanguera (Uniderp) in the city of Campo Grande, MS, Brazil, from April 2015 to November 2016.

Were used 41 male Norvergic Wistar rats, weighing 250 g-300 g, coming from Hospital Veterinário da Uniderp. The animals were kept in cages measuring 40 cm x 33 cm and 17 cm depth, with four rats per cage, at an ambient temperature of approximately 23ºC, with light cycles of 12 h and fed with Nuvital^(r)^ feed and water *ad libitum*. The research was authorized by the Ethical Committee on the Use of Animals of Anhanguera Educacional Ltda. - CEUA/AESA for the 2996 sight.

### Groups

The animals were distributed in the following groups:

- Group A - Ischemia and Reperfusion: nine rats were submitted to intestinal ischemia for 70 minutes by aortic clamping, followed by reperfusion for 70 minutes;
- Group B - Ischemic postconditioning: nine rats were submitted to the ischemia procedure for 70 minutes by aortic clamping and reperfusion for 70 minutes. Between ischemia and reperfusion, four cycles of reperfusion (30 seconds each) were performed, interspersed with four cycles of ischemia (30 seconds each);
- Group C - Ischemic postconditioning + Statin: nine rats received a dose of 3.4 mg/day of atorvastatin, one dose per day through the gavage method, for seven days and underwent the ischemia procedure for 70 minutes per aortic clamping and reperfusion for 70 minutes. Between ischemia and reperfusion, four cycles of reperfusion (30 seconds each) were performed, interspersed with four cycles of ischemia (30 seconds each);
- Group D - Statin: nine rats received a dose of 3.4 mg/day atorvastatin, one dose per day through the gavage method, for seven days and underwent the ischemia procedure for 70 minutes by aortic clamping and reperfusion for 70 minutes;
- Group E - SHAM: five rats submitted only to laparotomy and manipulation of the abdominal aorta.

### Anesthesia

The anesthesic procedure used in the experiment was an intraperitoneal injection of 2:1 solution with ketamine hydrochloride (Cetamin^(r)^), 50 mg/mL, and xylazine hydrochloride (Xilazin^(r)^), 20 mg/mL, respectively, at the dose of 0.1 mL/100 g)^[10]^.

### Surgical Procedure

The rats were submitted to a median longitudinal laparotomy of approximately 4 cm, exteriorization of the small intestine, identification and dissection of the abdominal aorta.

In all groups except SHAM, the abdominal aorta was occluded by atraumatic vascular clamp that remained for 70 minutes (ischemia phase).

After clamp placement, the small intestine was repositioned into the abdominal cavity and the surgical wound was closed with continuous skin suture with 4-0 monofilament nylon (Mononylon^(r)^) yarn.

After the ischemia phase, the abdominal wall was reopened by removal of the suture and in groups A and D, the vascular clamp was removed, initiating the reperfusion phase, with a duration of 70 minutes. In groups B and C, preceding the reperfusion phase, the ischemic postconditioning was performed through four cycles of reperfusion (removal of the atraumatic vascular clamp from the abdominal aorta) with duration of 30 seconds each, interspersed with four cycles of ischemia (occlusion of the abdominal aorta artery by atraumatic vascular clamp), also with duration of 30 seconds each.

In all groups after the start of the reperfusion phase, the abdomen was again closed by continuous suturing of the skin with 4-0 monofilament nylon suture until the end of the experiment.

In group E (SHAM), only the median longitudinal laparotomy of approximately 4 cm was performed, with exteriorization of the small intestine, identification and dissection of the abdominal aorta.

After reperfusion phase, in groups A, B and C, animals of all groups underwent median thoracotomy and resection of the inferior lobe of the right lung and a segment of approximately 1 cm of the ileum, 5 cm proximal to the ileocecal transition, which was opened at its antimesenteric border; partial hepatectomy, covering the median and left liver lobes and total withdrawal of the direct kidney. After the procedure, all organs were washed with saline solution and placed in 10% formaldehyde solution for further histological analysis.

### Euthanasia

The euthanasia of the animals used in the experiments was by intraperitoneal administration of lethal dose of anesthetic hydrochloride ketamine + xylazine (0,4 mL per 100 g).

### Histopathological Study

The specimens were kept for 48 hours in formaldehyde solution, after which they were subjected to paraffin inclusion and cut into the micrometer to obtain the histological slides. All slides were stained with hematoxylin-eosin (general morphology of the organs) and analyzed by the pathologist under optical microscopy, without previous knowledge about the group belonging to each rat.

**Fig. 3:**
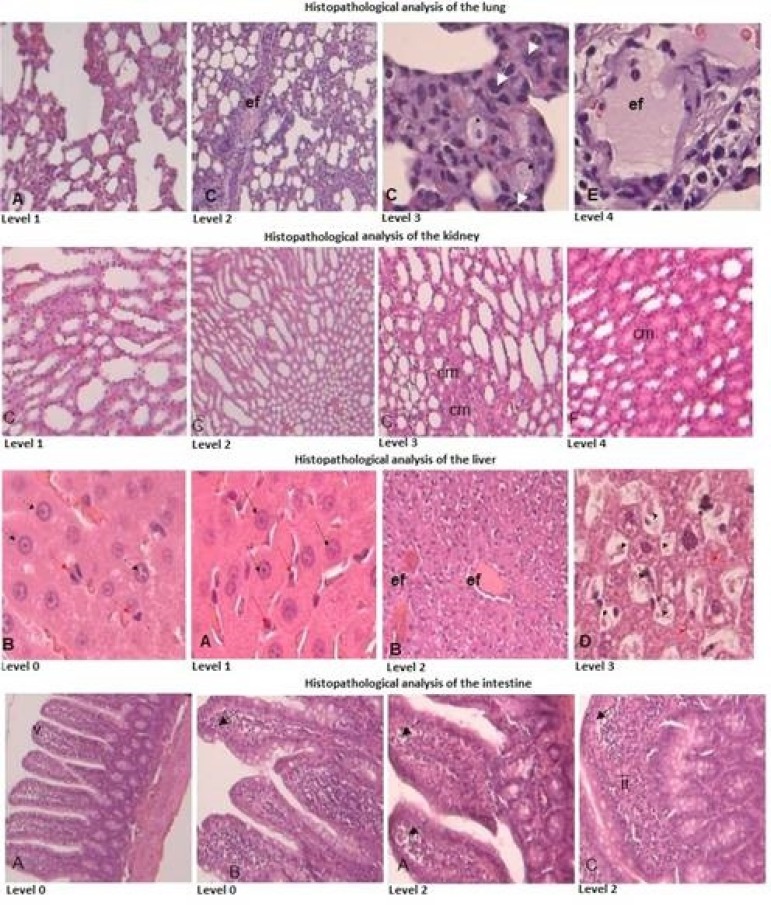
Histopathological analysis of lung, kidney, liver and intestine.

The pulmonary segments were classified according the degree of tissue injury from Greca et al.^[11]^ (Figure 3):

- Level 1 (normal): normal parenchyma under optical microscopy;
- Level 2 (mild): focal edema in few alveolar septum, mild congestion, neutrophils in alveolar septum in less than 50 per large increase field;
- Level 3 (moderate): moderate edema in alveolar septum or mild edema in several septum, moderate congestion, neutrophils in alveolar septum between 50 and 100 per large increase field;
- Level 4 (severe): severe edema in alveolar septum or mild edema in several septum, moderate congestion, neutrophils in alveolar septum more than 100 per field.

The right kidney was analyzed according to the presence of tubular necrosis, hydropic degeneration, medullary congestion, dilatation and tubular atrophy, following the scale developed by Shih et al.^[12]^, who considered (Figure 3):

- Level 0 = normal kidney;
- Level 0.5 = small focal areas;
- Level 1 = involvement of less than 10% of the renal cortex;
- Level 2 = involvement of 10-25% of the renal cortex;
- Level 3 = involvement of 25-75% of the renal cortex;
- Level 4 = involvement of more than 75% of the renal cortex.

The hepatic tissues were evaluated and quantified the ischemia and reperfusion injuries on the following histological characteristics: vascular congestion (sinusoidal, centrolobular and portal space), necrosis and hepatic steatosis.

The intensity of the histopathological features were expressed in crosses (0+ to 3+), obtained through the average of three random microscopic fields, being evaluated in a 200fold increase, considering the following graduation described by Rhoden et al.^[13]^ (Figure 3):

- Level 0+: absence of changes.
- Level 1+ = changes of mild intensity (less than 25% of the field analyzed);
- Level 2+ = changes of moderate intensity (25 to 50% of the field analyzed);
- Level 3+ = changes of severe intensity (more than 50% of the analyzed field).

The intestinal segments were classified according to degree of tissue injury from Chiu et al.^[14]^:

- Level 0 = mucous membrane without changes;
- Level 1 = well-constituted villi, without cell lysis or inflammatory process, but with formation of the Grunhagen subepithelial space;
- Level 2 = presence of cell lysis, formation of Grunhagen subepithelial space and increased spacing between the villi;
- Level 3 = destruction of the free portion of the villi, presence of dilated capillaries and inflammatory cells;
- Level 4 = structural destruction of the villi, with only a few sketches, consisting of inflammatory cells and necrotic material, with hemorrhage and basal glandular ulceration;
- Level 5 = destruction of all mucous membrane, no more glandular structure, but only amorphous material deposited on the submucosal screen.

### Statistical Analysis

The results were submitted to statistical analysis, applying the Kruskal-Wallis test, establishing the level of significance of *P*<0.05.

## RESULTS

### Histopathological Analysis of the Lungs

**Fig. 4:**
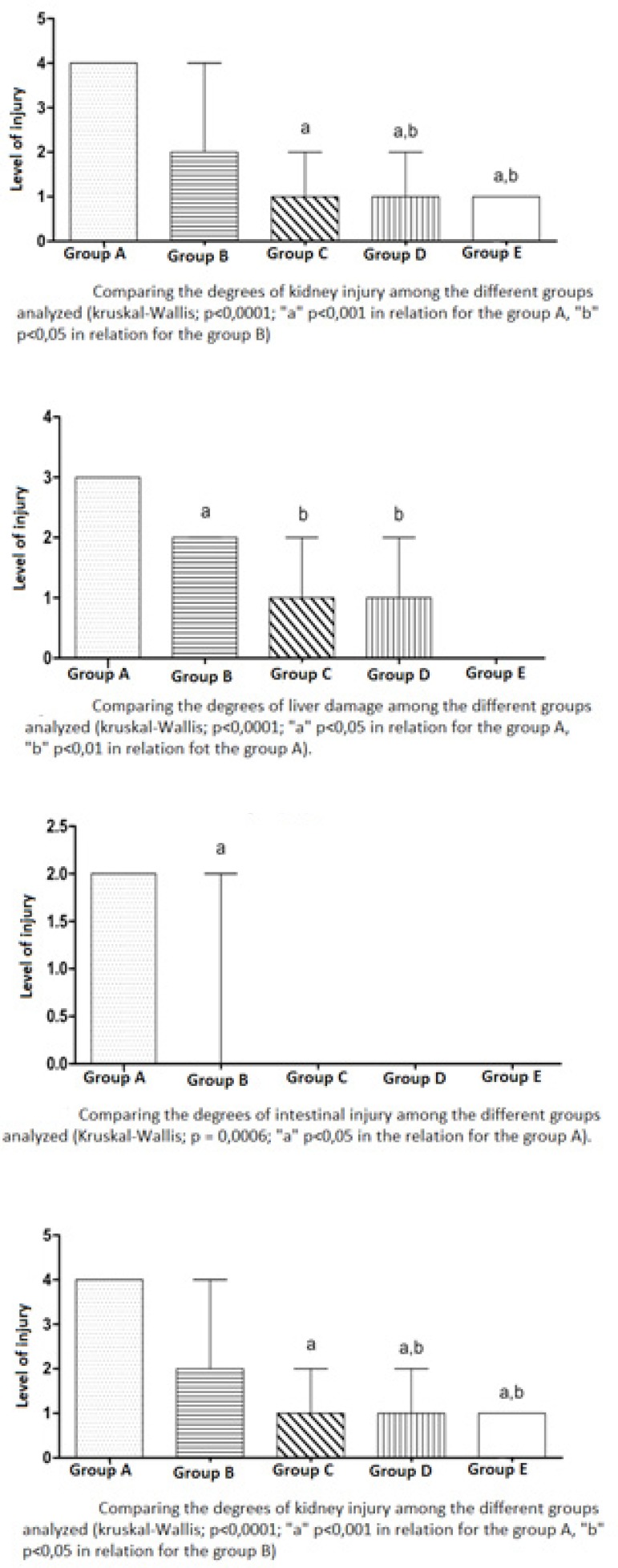
Graphics analyzing the level of injury among tissues in each organ.

The histopathological analysis of the pulmonary parenchyma specimens, according to Greca et al.^[11]^, showed mean values of 3.6 in group A; 1.6 in group B; 1.2 in groups C and D and 1 in group E (Table 1 and Figure 4).

**Table 1:** Results of statistic analysis of tissue injuries.

**Histopathological classification of lung parenchyma injury in rats**					
**Rats**	**A**	**B**	**C**	**D**	**E**
1	4	1	1	1	1
2	4	1	1	1	1
3	4	2	2	2	1
4	4	1	1	2	1
5	4	3	1	1	1
6	3	2	2	1	-
7	2	1	1	1	-
8	4	1	1	1	-
9	4	2	1	1	-
AVERAGE	3.6	1.6	1.2	1.2	1
*P*=0.0029 (Kruskal-Wallis)					
**Histopathological classification of liver parenchyma injury in rats**					
**Rats**	**A**	**B**	**C**	**D**	**E**
1	3	2	1	1	0
2	3	1	1	1	0
3	3	1	2	2	0
4	3	2	1	2	0
5	3	2	1	1	0
6	3	1	2	1	-
7	3	2	1	1	-
8	3	1	1	1	-
9	3	2	1	1	-
AVERAGE	3	1.5	1.2	1.2	0
*P*<0.0001 (Kruskal-Wallis)					
**Histopathological classification of intestinal parenchyma injury in rats**					
**Rats**	**A**	**B**	**C**	**D**	**E**
1	2	0	0	0	0
2	2	0	0	0	0
3	2	0	0	0	0
4	2	2	0	0	0
5	2	2	0	0	0
6	2	0	0	0	-
7	2	0	0	0	-
8	2	2	0	0	-
9	2	0	0	0	-
AVERAGE	2	0.66	0	0	0
*P*=0.0006 (Kruskal-Wallis)					
**Histopathological classification of renal parenchyma injury in rats**					
**Rats**	**A**	**B**	**C**	**D**	**E**
1	4	2	1	1	1
2	4	2	1	1	1
3	4	2	1	1	1
4	4	4	2	1	1
5	4	2	1	1	1
6	4	2	1	2	-
7	4	4	2	1	-
8	4	2	1	1	-
9	4	2	1	1	-
AVERAGE	4	2.44	1.22	1.11	1
*P*=0.0001 (Kruskal-Wallis)					

### Histopathological Analysis of the Kidney

In the histopathological analysis of the kidney specimens, a mean degree of injury 4 was found in group A, meaning involvement of more than 75% of the renal cortex; group B obtained a mean of 2.44; C group obtained a mean of 1.22 and in group D the mean D was 1.11. Group E obtained mean 1 (Table 1 and Figure 4).

### Histopathological Analysis of the Liver

Among the histopathological analysis of the liver was used the classification of Rhoden et al.^[13]^ on the 41 slides.

It was concluded from the analysis that group A obtained a 3+ mean of injury, being considered intense alterations; group B obtained a mean of 1.5+; groups C and D obtained the same means of degree of injury: 1.2 + (Table 1 and Figure 4).

### Histopathological Analysis of Intestin

In the analysis of the intestine slides, pathological changes were observed in group A of ischemia and reperfusion and a protective action of the treatments.

Group A, that underwent ischemia/reperfusion, obtained average degree of injury 2; group B associated with ischemic postconditioning obtained a mean of 0.66. The other groups, C, D and E, obtained degree 0 of injuries, which means that there were no alterations (Table 1 and Figure 4).

## DISCUSSION

In recent years, research on oxidative stress has been increasingly important because it is the inflammatory pathophysiological mechanism that determines many chronic degenerative diseases, such as heart failure, central nervous system diseases, oncogenetics, among others, and acute events with a high rate of mortality, such as multiple organ dysfunction syndrome^[15,16]^.

Thirty years have passed since Murry et al.^[3]^ proposed the technique of ischemic preconditioning. Because it is a procedure with limitations, due to the impossibility of employing it in several situations, justified by the fact that ischemia is already installed, new alternatives were sought, among them ischemic postconditioning.

In several clinical situations, it is not possible to use any method, since the organ artery is not accessible. Brief episodes of ischemia/ reperfusion were applied to remote tissues, such as small intestine, kidney, liver and even lower limbs, and demonstrated to reduce infarct size in hearts submitted to coronary occlusion, followed by reperfusion - a technique called remote preconditioning.

Dorsa et al.^[5]^, in a study involving 3 groups of rats, one control group, and two groups submitted to ischemia/reperfusion by abdominal aorta clamping, in one of which the remote postconditioning technique was used. As in the present study, these researchers used Greca et al.^[11]^ classification to evaluate the degree of lung parenchymal injury. The result obtained in the Dorsa's study is very similar to the result of the present study, since the mean of the degree of lung injury was 1.3 and in this study was 1.6 when it refers to the post-ischemic conditioning^[5]^.

In a work carried out by Naidu et al.^[17]^, mice were treated with simvastatin for five days, lungs submitted to ischemia for 90 minutes and reperfusion for 4 hours, demonstrating a 71% reduction in pulmonary vascular permeability when compared to the untreated group. In addition, there was a 68% reduction of tissue myeloperoxidase and reduction of leukocyte accumulation in bronchoalveolar lavage. In the present study, the group treated with atorvastatin for seven days presented preservation of the pulmonary parenchyma, absence of hemorrhagic focus, congested blood vessels, necrosis, neutrophil accumulation and focal edema. It presented only inflammatory focus, showing a significant reduction of the lesion in comparison to group A (positive control), whose grade was 3.6. According to Greca et al.^[11]^, in the present study, the degree of injury in groups C (atorvastatin only) and D (atorvastatin and ischemic postconditioning) was 1.2 in both groups.

In the study by Sun et al.^[18]^, in rats submitted to lower limb ischemia for two hours, followed by three hours of reperfusion, the groups receiving statin pretreatment for three days also demonstrated reduction of myeloperoxidase and malondialdehyde levels in tissues, neutrophil accumulation and significant decrease in lung parenchymal lesions compared to the group that underwent ischemia and reperfusion without previous treatment, according to the histopathological findings of the present study^[18]^.

The literature does not have yet works involving the histological analysis of the use of remote postconditioning in the prevention of ischemia/reperfusion injury at a distance, especially in organs used in the present study, such as liver and kidney.

Seifi et al.^[19]^ studied the degree of hepatic impairment in three groups of rats: a control group, a group submitted to ischemia/reperfusion of the left renal artery and one group submitted to ischemia/reperfusion and protected by remote ischemic postconditioning performed in the left renal artery. The results were analyzed by the biochemical assay and by the measurement of oxidative stress markers. The postconditioned group showed a significant decrease in alanine transaminase (ALT) and aspartate transaminase (AST) liver injury enzymes in relation to the ischemia/reperfusion group, and a lower dosage of malondialdehyde^[19]^. In the present study, remote postconditioning also had a protective effect on the hepatic parenchyma, with the mean degree of injury 1.5+ according to Rhoden et al.^[13]^, and in contrast to the mean of 3+ in the group submitted to ischemia/reperfusion alone.

In the experimental research carried out by Lai et al.^[20]^, the group in which pharmacological preconditioning was performed with intraperitoneal simvastatin demonstrated a significant reduction in the levels of liver injury enzymes, ALT and AST, and reduction of apoptosis of hepatocyte, compared to the group submitted to ischemia/reperfusion of the liver without treatment. In the present study, we observed reduction of vascular congestion, vessel thickening, necrosis and apoptosis of hepatocytes and tissue, centrolobular edema. Quantitatively, the degree of injury, according to Rhoden et al.^[13]^, of the group undergoing pretreatment with atorvastatin presented the mean of 1.2, contrasting with the mean of 3 of group A (positive control). The group that was associated with atorvastatin with ischemic postconditioning presented the same protection and the same mean of the degree of injury of 1.2.

The literature does not yet have experimental work that tests the pretreatment with statin in the remote ischemia/reperfusion lesion, and studies that use the histopathological analysis to quantify the degree of injury and the protection capacity of this method. For this reason, the results found in the present study cannot be adequately compared. However, in the studies already published and in the present study, statin has been shown to be a promising drug in the prevention of ischemia and reperfusion injury and in acute oxidative stress.

## CONCLUSION

The groups submitted to postconditioning, pretreatment with statins and the two associated methods (groups B, D and C, respectively) showed a lower degree of ischemia and a remote reperfusion injury compared to the group submitted to I/R injury without protection (group A), and the groups treated with statins showed slightly better results than the postconditioning group. Further studies regarding the use of statins and postconditioning should be performed to elucidate its mechanisms and ratify its effectiveness in the prevention and minimization of ischemia and reperfusion lesions.

**Table:** 

Authors' roles & responsibilities	
HBDP	Substantial contributions to the conception or design of the work; or the acquisition, analysis, or interpretation of data for the work; final approval of the version to be published
JCDVP	Substantial contributions to the conception or design of the work; or the acquisition, analysis; final approval of the version to be published
EAN	Substantial contributions to the conception or design of the work; or the acquisition, analysis; final approval of the version to be published
GSCV	Agreement to be accountable for all aspects of the work in ensuring that questions related to the accuracy or integrity of any part of the work are appropriately resolved; final approval of the version to be published
JVCM	Agreement to be accountable for all aspects of the work in ensuring that questions related to the accuracy or integrity of any part of the work are appropriately and resolved; final approval of the version to be published
LESD	Agreement to be accountable for all aspects of the work in ensuring that questions related to the accuracy or integrity of any part of the work are appropriately and resolved; final approval of the version to be published
JVDGO	Agreement to be accountable for all aspects of the work in ensuring that questions related to the accuracy or integrity of any part of the work are appropriately and resolved; final approval of the version to be published
MHMA	Agreement to be accountable for all aspects of the work in ensuring that questions related to the accuracy or integrity of any part of the work are appropriately and resolved; final approval of the version to be published
IOC	Agreement to be accountable for all aspects of the work in ensuring that questions related to the accuracy or integrity of any part of the work are appropriately and resolved; final approval of the version to be published
TLS	Agreement to be accountable for all aspects of the work in ensuring that questions related to the accuracy or integrity of any part of the work are appropriately and resolved; final approval of the version to be published
CHMS	Substantial contributions to the conception or design of the work; final approval of the version to be published
DMD	Substantial contributions to the conception or design of the work; or the acquisition, analysis; drafting the work or revising it critically for important intellectual content; final approval of the version to be published
